# Use of the Dynamic External Distractor Ligamentotaxor in finger trauma

**DOI:** 10.1186/1753-6561-9-S3-A49

**Published:** 2015-05-19

**Authors:** Michel Schoofs

**Affiliations:** 1 SOS Main Lille, Lesquin, 59810 France

## Introduction

The treatment of fracture dislocations of the fingers is difficult.

The concept of capsuloligamentotaxis is familiar but the systems of external distraction are cumbersome.

The Suzuki system is ingenious but not devoid of complications.

## Materials

We report results of a series of 35 fingers in 32 patients treated by the Ligamentotaxor® external distraction system.

## Results

Twenty-five patients were operated in emergency following complex MCP, PIP and DIP fractures and 7 patients at more than one month following trauma due to neglected dislocation or complications of arthroplasty.

The duration of distraction was 6 weeks.

Associated osteosynthesis was performed in 3 cases. The mean follow-up was 36 months.

Grip strength was 85% of the contralateral side.

Active flexion was 75° and flexion deformity 20°.

Pain recorded using the VAS was 1.5 and quick DASH score was 17.

One patient had sepsis requiring premature removal of metalwork.

One patient disassembled the distraction system himself.

Bone union occurred in all cases.

## Conclusions

Despite the imperfections of the results, we think that Ligamentotaxor® is not cumbersome, easy to assemble and useful in treatment of complex PIP finger fractures as well as other fractures or sequelae of dislocations and arthroplasty based on an effect of capsuloligamentous, tendinous and skin taxis.

